# Preparing to “Live a Life of Possibilities”: Experiences of Healthcare Providers Readying Autistic Adolescents and Their Families for Independent Driving

**DOI:** 10.1007/s10803-024-06335-0

**Published:** 2024-04-25

**Authors:** Rachel K. Myers, Christina Labows, Catherine C. McDonald, Benjamin E. Yerys, Emma B. Sartin, Meghan E. Carey, Cynthia J. Mollen, Allison E. Curry

**Affiliations:** 1 Center for Injury Research and Prevention, Children’s Hospital of Philadelphia, 2716 South St, 13th Floor, Philadelphia, PA 19146 USA; 2 Division of Emergency Medicine, Department of Pediatrics, Perelman School of Medicine, University of Pennsylvania, 3401 Civic Center Blvd, Philadelphia, PA 19104 USA; 3 Department of Family and Community Health, School of Nursing, University of Pennsylvania, 418 Curie Blvd, Philadelphia, PA 19104 USA; 4 Division of Pediatrics, Department of Pediatrics, Perelman School of Medicine, University of Pennsylvania, 3401 Civic Center Blvd, Philadelphia, PA 19104 USA; 5 Department of Psychiatry, Perelman School of Medicine, University of Pennsylvania, 3400 Civic Center Blvd, Philadelphia, PA 19104 USA; 6 Center for Autism Research, Children’s Hospital of Philadelphia, 2716 South St, 5th Floor, Philadelphia, PA 19146 USA; 7 PolicyLab, Children’s Hospital of Philadelphia, 2716 South St, 10th Floor, Philadelphia, PA 19146 USA

**Keywords:** Autism spectrum disorder, Adolescent health, Transportation, Licensure, Parents, Healthcare providers

## Abstract

Autistic adolescents and their families may experience barriers to transportation, including independent driving, which is critical to supporting quality of life and engagement in social, educational, and employment opportunities. Healthcare providers may feel unprepared to provide guidance to autistic adolescents, although they are among the professionals families turn to for guidance. This study describes providers’ experiences supporting autistic adolescents and families in the decision to pursue licensure and identifies barriers experienced in providing support. We conducted interviews with 15 healthcare providers focused on how they support autistic adolescents and their families in navigating topics related to independence, driving, and transportation. Key themes identified included: importance of understanding adolescents’ perspectives and motivations, approaches to readying caregivers for children to pursue driving, and role of providers in fostering agreement between adolescents and caregivers. Results reflect healthcare providers as intermediaries between autistic adolescents and caregivers making the decision to pursue licensure and bring families to consensus. Our findings emphasize the importance of healthcare providers, in collaboration with community-based providers, in supporting autistic adolescents and their families considering licensure. Improving conversations between providers and families provides opportunity to better support quality of life among autistic adolescents and their caregivers navigating the transition to independence.

For autistic adolescents, the transition to adulthood can be hampered by limited access to supportive services, as well as caregivers’ and other professionals’ lack of preparation to support development of independent life skills. Despite evidence that specialized support greatly enhances quality of life and community integration, recent national surveys of high-school and transition-aged autistic adolescents found that 25% of autistic adolescents received no transition support services during the critical transition from adolescence to early adulthood (Mason et al., 2018; Myers et al., 2015; Roux et al., 2015). Of particular concern during autistic adolescents’ transition to adulthood are challenges related to daily living skills including transportation access, which is critical to supporting continued engagement in social, educational and employment opportunities throughout emerging adulthood. While many autistic adolescents and their caregivers express interest in becoming licensed to drive independently, characteristics of autism, including difficulties with executive functioning, social communication, cognition, and emotional reactivity, may present the need for specialized planning and support to help build the skills required for driving (Havlicek et al., 2016; Huang et al., 2012; Kirby et al., 2020).

One-third of autistic adolescents experience challenges in travelling to places outside their homes or are prohibited from doing so independently (Shattuck et al., 2018). Autistic adolescents and their families appear to face challenges in navigating the path to licensure, as evidenced by high interest in driving among autistic adolescents but only one in three obtaining their license before age 21 (Curry et al., 2018). Lack of access to independent transportation may contribute to feelings of isolation and depression among adolescents and create logistical and financial hardships for caregivers, who are often the only source of their child’s transportation access (Deka et al., 2016; Zalewska et al., 2016). Further, caregivers report challenges accessing supportive services and feel unprepared to assist their children in preparing for independence (Cheak-Zamora et al., 2015). Given this, families who are considering whether to pursue driving may seek guidance from healthcare providers to assess driving readiness and identify opportunities to foster skills critical to licensure and safe driving. Autistic adolescents receive health care from diverse teams of providers—including but not limited to physicians, advanced practice professionals, psychologists, and social workers who may provide continuous care over a period of years and are trusted resources for anticipatory guidance regarding transition to adulthood (Cummings et al., 2016; Kuhlthau et al., 2016).

In a prior quantitative survey of healthcare providers, we found that providers feel unprepared to provide transition guidance to autistic patients, including guidance related to transportation such as driving, resulting in delayed information sharing and diminished time for effective transition planning (Myers et al., 2022; Sartin et al., 2021). Building on this prior research, we conducted a qualitative study with diverse healthcare providers to elucidate their experiences working with autistic adolescents and their families preparing for independent driving and to gain insight to their perceived resource needs to support adolescents and families. The objectives of this study are to (1) describe providers’ perspectives of their role in supporting autistic adolescents and caregivers in the decision to pursue driving and (2) identify specific barriers providers experience in providing this guidance and support. These results will help to inform efforts to improve transportation-related anticipatory guidance provided to autistic adolescents and their families in the healthcare setting.

## Methods

### Design and Participants

We conducted semi-structured interviews with healthcare providers with self-reported experience providing transition-related care to autistic adolescents. Healthcare providers who expressed interest in participating in a qualitative interview following completion of a brief electronic survey (Myers et al., 2022; Sartin et al., 2021) or who were referred by word of mouth were invited to participate. All participants provided care to autistic adolescents without intellectual disabilities and were employed at the Children’s Hospital of Philadelphia or the Philadelphia Autism Centers of Excellence. The study was determined to be exempt of oversight by the Children’s Hospital of Philadelphia Institutional Review Board (Approval #18-015740) with a waiver of written documentation of consent.

We conducted interviews with 15 healthcare providers, the majority of whom had five or more years of clinical practice experience with autistic adolescents (Table 1). Participants represented a diversity of roles including physicians, psychologists, social workers, and nurse practitioners. Data regarding socioeconomic status were not collected.

**Table 1:** Participant characteristics (N = 15)

Characteristics	N (%)
Female	13 (86.7%)
*Race*	
Black or African American	3 (20.0%)
White	12 (80.0%)
Hispanic or Latino	1 (6.7%)
*Years in practice*	
0–4 years	5 (33.3%)
5–10 years	4 (26.7%)
11–15 years	0 (0%)
16–20 years	1 (6.7%)
> 20 years	5 (33.3%)
*Current clinical role*	
Physician	6 (40.0%)
Nurse practitioner	1 (6.7%)
Psychologist	4 (26.7%)
Social worker	3 (20.0%)
Therapist	1 (6.7%)

### Interviews

We prepared an interview guide informed by the study team’s prior experience conducting qualitative research with specialized driving instructors regarding the learning-to-drive process for autistic teens (Myers et al., 2019, 2021) and a comprehensive literature review on both driving and non-driving transportation use among autistic individuals.

Interview questions explored how providers offer guidance to autistic patients on topics related to driving and transportation. These included (1) experiences providing guidance to parents and autistic adolescents regarding driving and licensure, (2) challenges and barriers encountered during these conversations, (3) discussion of autistic adolescents’ perceived strengths, (4) concerns providers have regarding autistic adolescents’ ability to drive, (5) experiences providing guidance about non-driving transportation, and (6) resources providers used and believe would be helpful.

The single study interviewer (CL) trained with the team’s qualitative experts (CJM and RKM), both of whom have extensive training and experience in qualitative research methods. The interviewer conducted multiple mock interviews to ensure questions were clear and to gain familiarity with the interview guide. Interviews were conducted over the phone, audio-recorded, and transcribed. The interviewer took supplementary notes to provide clarity for any concerns with transcription and to help identify when thematic saturation had been achieved. The first four transcripts were reviewed by CJM and RKM to ensure that interview questions were clear to participants and to provide feedback to the interviewer. Following this review, interview data were reviewed at regular intervals to address any emergent concerns or difficulties. No participants dropped out of the study or terminated interviews early. Interviews lasted a mean of 30 min, ranging from 21 to 38 min. All participants received a $20 gift card as remuneration for their participation.

### Data Analysis

All interviews were transcribed and de-identified before importing into NVivo (Version 11, QSR International) for organization and analysis. The study team met routinely to discuss topics arising in interviews and determine when the interviews achieved thematic saturation. Using a content analysis approach, we developed a codebook based on previous literature and the interview guide. We selected four transcripts to be double coded by the interviewer (CL) and a secondary coder (LL, graduate student research assistant). Both coders received training in qualitative coding from a member of the research team (RKM). Two team members independently coded each transcript, assessed inter-relater reliability via Cohen’s K, and discussed any discrepancies in coding. When discrepancies were not resolved between the two coders, two members of the research team (MC and RKM) were consulted to resolve coding questions. After coding nine transcripts, the coders demonstrated consistent application of the codebook, and the primary coder completed coding for the remaining six transcripts. After all transcripts were coded, the inter-rater reliability demonstrated a mean K = 0.87 with a range of 0.82–0.97. Five members of the research team (CJM, BEY, RKM, CM, CL) with qualitative or clinical expertise read the coded text segments and prepared thematic summaries. Discussion of the summaries of the coded text led to the results presented in this manuscript.

### Community Involvement

Regarding community involvement, two practitioners with expertise in driving and transition to adulthood for autistic adolescents reviewed the interview guide prior to initiating data collection. The first was an Occupational Therapist and Licensed Driving Instructor (LDI) who specializes in teaching autistic adolescents to drive and the second was a PhD-level researcher whose research addresses the transition to adulthood for individuals with neurodevelopmental differences. These practitioners were external to the research team and reviewed drafts of the interview guide. Their review helped to ensure clarity and appropriateness of question wording.

## Results

### Interview Themes

*Understanding Adolescent Perspectives* Providers highlighted their efforts to understand adolescents’ own perspectives, motivations, and perceived readiness for licensure and driving. Several described how autistic patients expressed a high degree of motivation to obtain their drivers’ license in part to obtain independence “so that they don’t feel like they’re a child forever and….they’re doing the same type of things that their peers are doing.” Providers perceived many adolescents viewed licensure as an important developmental milestone enabling them to become more independent, noting: “A very high level of motivation to be more independent is something that I see a lot. They really crave that independence, and they know driving would allow for that. And so they’re more motivated than some…typically developing teenagers.”

While motivation to become independent was widely described, providers discussed the importance of understanding adolescents’ specific motivation to obtain their license, which often pertained to quality-of-life concerns, such as continuing their education, facilitating employment, or maintaining social connections. One participant described “If it’s ‘I need to get to work …there are things I want to do in my life that I need to have …be able to drive to be able to access’ – whether that’s social relationships, education, work…that’s usually what…our conversation is tied around.” Equally as important for several providers was understanding the perspective of adolescents who felt unready or uncertain about driving. Providers described that for many adolescents, driving-related anxieties included concerns ranging from understanding behind-the-wheel social cues to managing potential crashes and interacting with police. Specifically related to the social elements of driving, one participant described: “It was a conversation around what are some of the reasons you’re uncomfortable, and a lot of them actually had to do with…’I don’t read social cues right, so what if I don’t read the ones on the road right, they might have really serious consequences.’”.

*Readying Caregivers for Driving* Many providers described interactions with and support provided to caregivers in preparation for their child pursuing licensure. Specifically, they recounted conversations focused on preparing caregivers to support their child’s increasing independence with one provider telling caregivers, “yes, your child may have a diagnosis…but they are…going to start to transition into adulthood, and they should have the same opportunities to do the things that other teens and other young adults have.”

This process of helping caregivers relinquish longstanding expectations and experiences of control in their relationships with their children was difficult for many providers to navigate. Participants described how caregivers seemed unprepared for their child’s growing independence, with driving being a particularly challenging transition topic. One provider compared this parental transition to a grieving process, sharing how one mother was:

having a really hard time letting him do anything that would move him towards autonomy and being an autonomous adult…Like a balance between keeping them safe and protected, and letting them have that freedom and feeling like they have a sense of self…control over what they choose to do. I think that’s a tough balance for this population’s parents to strike. And driving’s like the perfect issue in terms of that balance, because driving is something that could really put them in harm’s way. And they could mess up, and they could get in a terrible accident, but they also need the opportunity to be autonomous. And I think like working through that like grieving process, and trying to think of like a different way of parenting, that they didn’t expect to have to do.

While many participants focused on adolescents’ independence related to licensure and driving, they noted caregivers were undergoing an important transition themselves regarding their relationship with their child, while managing their own set of concerns about their child’s ability to safely drive.

*Navigating Beliefs and Fostering Agreement* Providers described guiding a range of families, from those who had not yet considered licensing and independent driving to those who had already made plans and identified supportive resources on their own. One provider noted that prior to their work with a family, “there wasn’t anyone ushering the family through [the decision to drive]. And it was looming, he was almost 16 years old and wanting to drive and no one had ever dealt with this.” Additionally, providers shared encounters in which they were forced to address mismatches between adolescents’ and their caregivers’ expectations for driving and independent transportation. One noted: “the family went ahead and decided that they were going to get him his permit, but now they’re facing a lot of difficulty as they try to get him to drive because he doesn’t actually feel comfortable driving while the family does.” Providers discussed how mismatched expectations led to violence, disagreements, emotional outbursts, and disappointment, making the provider’s role of offering guidance and support difficult at times.

When providers believed that licensure was an appropriate and feasible goal, providers tried to help adolescents and caregivers view licensure and driving through a lens of the opportunities for independence and improving quality of life it could offer, with one provider sharing:

There’s so many benefits [of] being able to drive that far outweigh the risks. And that’s part of the conversation I have with parents. Look, I know that you’re scared, but if he can do this, this is gonna be amazing for your family, right, like, you can go on vacation and he can actually live his life without you and be fine….But I try and frame that like, this is a positive for your whole family, that they can be more independent. But it’s also a huge positive for [your child], because now they can feel like they’re not a child anymore. Like they’re starting to really, truly feel like an autonomous adult. And that’s ultimately what we want. We want them…to live a life of possibilities completely autonomous and independent as possible, the least restrictive environment. And driving is part of that.

Providers also perceived that some adolescents encountered difficulties with this emerging independence from their caregivers and perceiving a sense of self-determination: “It’s also maybe something, where for that adolescent they’re used to their parents being their caregivers, doing things for them. Where [driving] is something that you’re doing on your own and you have to have that full control.”

For adolescents for whom the decision to pursue licensure was appropriate, providers frequently discussed working to address the previously described anxieties related to driving, with one sharing: “[I] kind of just talk…how to make the decision of whether or not his discomfort is a valid concern or if it’s related to a different diagnosis of anxiety and eventually getting to the point of talking about some of this sounds like really reasonable concerns that all teenagers have about driving and not just you and really weaving it to be a family discussion that I can guide with some questions.” Providers recognized that each patient required an individualized approach to address their specific needs; for some their needs focused on physical readiness (e.g., motor control and coordination), while for others their needs centered on social and emotional readiness.

Providers believed that licensure and driving readiness were often part of a continuum to independence and their role was to “allow… or help… families realize that your teen can do these things and can have [this] – level of independence and how do you work together to get to that point.” One participant further described how becoming licensed to drive was an important developmental milestone for autistic adolescents as it is connected with “goal setting and goal achieving…this idea of setting a goal and achieving and celebrating … it resonates more [with autistic adolescents].”

*Barriers Experienced in Preparing Adolescents and Families* Providers’ efforts to prepare and discuss the sensitive and complex decision to pursue licensure and independent driving was not without challenges. Providers described feeling unprepared to effectively support adolescents and families in navigating decisions around licensure and driving readiness on their own, noting “if there are not appropriate resources and…if there’s not back up that clinicians can use to help families through this, then it’s a very tricky thing to be able to deal with in a quick clinic visit. And it’s potentially a very high stakes thing to talk about.” Specifically, several providers wished they knew in advance when these discussions might occur, so that they could be prepared or collect collateral information from others with knowledge of the patient, such as teachers and therapists. Additionally, providers expressed a desire for better knowledge or understanding of available community-based resources to which they could refer families, with some describing locating resources for families through their own internet searches or informal word of mouth from colleagues. One noted: “I think there are not sufficient – or not easily accessible for my patients – driver evaluations to make sure that the patient has the skill set necessary to learn to drive. So I think more programming would reduce that sort of barrier that sometimes – because again, I had to go online and find one in [County], and do a little calling and find out that it seemed to be appropriate.”

Further, providers described how limited appointment time impeded their ability to effectively counsel and prepare adolescents and their caregivers both separately and together:

I think the top barrier is just time because… we need to cover all of their regular med management topics, and then usually driving is an afterthought, so having time to get the conversation in is usually the hardest part. And then in the conversation it can be really hard because it’s oftentimes the first time anybody has sat down and talked about this all together – so either not having both parents there or having both parents on different pages, having the kid trying to turn it into an I-told-you-so, I-told-you-so, and their parents are like, whoa, whoa, we weren’t ready for this. Those tend to be the biggest barriers of this is the first time we’ve ever talked about it and not everyone is here or Mom and Dad haven’t talked about it together and don’t have a united front yet.

Providers further recognized that for some adolescents and their families, significant external factors existed, such as limited vehicle access, costliness, and safety, which were beyond providers’ ability to provide guidance to adolescents and caregivers. One provider described a common experience where a “family might only have one car and two parents and then a teen who wants to drive.” Regarding the potential costliness of obtaining specialized driving evaluation and instruction, one provider shared it would be helpful to know “how [a family] can get funding, because out-of-pocket driving lessons can be very expensive and that can be a barrier for families. So, if there were any type of special funding programs, any special agencies, that would be helpful to have, and I would give that out to families.”

## Discussion

Our results highlight the complexities experienced by healthcare providers as they navigate conversations between autistic adolescents and their families regarding licensure and independent driving. Licensure can be an important developmental milestone, representing a turning point in establishing autonomy and independence, and may be foundational in enhancing quality of life for both adolescents and caregivers, while supporting access to educational, vocational, and social opportunities (Lubin & Feeley, 2016; Zalewska et al., 2016). Healthcare providers perceived their role as an intermediary between understanding the motivations and concerns of adolescents and caregivers making the decision to pursue licensure and driving and helping to bring families to consensus. However, providers encountered barriers related to their own knowledge and time, as well mismatched expectations within families, which made providing counseling about licensure and driving more difficult. Our results highlight the importance of healthcare providers in supporting autistic adolescents and their families considering and readying for licensure and the opportunities to enhance providers’ ability to use such conversations to foster autistic adolescents’ independence.

Participants described the importance of understanding the perspectives of autistic adolescents in the decision to pursue licensure and independent driving. Both autistic adolescents and their caregivers previously have described a desire for independence, but they feel uncomfortable or uncertain of how to support and achieve such autonomy (Cheak-Zamora et al., 2017). Many caregivers of autistic adolescents report longstanding responsibility for meeting their child’s needs, especially related to transportation (Lubin & Feeley, 2016). Importantly, licensure and driving may bring into focus the tension between parental readiness to relinquish control and autistic adolescents’ emerging self-determination (Cheak-Zamora et al., 2020; Kim, 2019). Licensure and driving are concrete activities that reflect shifting expectations of both adolescents and caregivers, which are often based on longstanding behaviors and experiences. Listening to the concerns, desires, and motivations of autistic adolescents is vital to ensuring providers feel able to assess readiness, provide concrete actions to help prepare for driving, and recognize and respect adolescents’ self-determination. This approach highlights the importance of shifting autonomy and fostering autistic adolescents’ ability to engage in decision-making about their transition to adulthood. By understanding the motivation of adolescents to drive, providers felt better prepared to ready both adolescents and caregivers, building adolescents’ confidence and competence.

Although providers’ clinical care focused on their adolescent patients, participants believed a key part of their role was readying caregivers for their child’s transition to independence, with driving reflecting one step of that process. This is consistent with caregivers wish for their child’s providers to give guidance and information on transition topics, especially to address parental uncertainty around independence (Cheak-Zamora et al., 2017; Jones et al., 2021). Prior research established the critical role that caregiver expectations of autistic adolescents’ future outcomes have with regards to independence (Chiang et al., 2013; Kirby, 2016). Supporting families navigating this transition to independence is consistent with the role of providers in fostering caregiver-adolescent communication for other health topics, such as sexual health, substance use, and transition to adult care (Ford et al., 2019; Leung et al., 2021White et al., 2018). Existing data suggest only moderate agreement between autistic adolescents and their caregivers with regards to expectations for education, employment, and independent living (Kirby et al., 2022). Our results expand this understanding of discordance in expectations to include licensure and independent driving. With a family-focused lens, providers often assume the role of consensus builder, working to help caregivers recognize the changing nature of their relationship with their child throughout adolescence and into emerging adulthood and bring adolescents and their caregivers into alignment about future expectations.

Providers experienced several barriers, which contributed to feeling underprepared or limited in their ability to provide adequate or timely guidance. Participants reported wanting to feel more prepared for conversations with patients and caregivers, by knowing in advance when such conversations may arise, having time to obtain input from other providers familiar with the patient, and developing a better knowledge of community-based resources, such as driving training programs. Appointment time remained a significant barrier, as has been previously described in other areas of adolescent anticipatory guidance and transition planning (Jones et al., 2021; Schraeder et al., 2020). Our results also highlight that while adolescents and families may look to healthcare providers for guidance regarding readiness to drive, providers alone cannot provide the full range of support that families need to prepare for licensure and driving. Rather, multidisciplinary teams could be helpful with information gathering, assessment, referrals, and connection to community-based services that would allow providers to more comprehensively support patients and families. Such a model has been applied more widely in medical transition to adult care for adolescents with other special health care needs than for those with autism and there may be opportunities to replicate such approaches specifically for autistic adolescent patients (Cheak-Zamora et al., 2013; Todorow et al., 2018). Finally, opportunities to strengthen the relationship between healthcare providers and community-based services, such as certified driving rehabilitation specialists, may help to facilitate a warm handoff to resources that can be customized and tailored to autistic adolescents’ needs (Myers et al., 2019, 2021).

There are several limitations to this study. First, we interviewed healthcare providers in a single city, with the majority working within a single institution. Although our sample included both medical and mental health providers, there are providers whose experiences are not reflected in these results. Second, because of the voluntary nature of this study, we may have only elicited perspective of experienced providers or those with particular interest or expertise in this topic area. Providers with less experience are likely not represented and may perceive different barriers that are not represented herein. Our results may not be transferable to providers with more limited experience counseling patients and families regarding licensure and driving who may require different resources. Finally, our sample of providers had limited demographic heterogeneity, namely with regards to race and gender. Our results do not well capture the experiences of providers from minority racial groups and we did not explore the potential for how differences between provider and family racial or ethnic identity may influence these conversations and participants’ experiences of providing care.

## Conclusions

Our work reflects the important role that healthcare providers play in supporting adolescents and caregivers in navigating decisions regarding licensure and independent driving. However, not all adolescents or families will choose or be able to pursue driving. Therefore, future research is needed to examine the role of healthcare providers in conversations about supporting adolescents’ use of alternative forms of transportation to support independence and ensure continued participation in activities critical to supporting transition to adulthood and quality of life. Further, it is vitally important to listen to the experiences of adolescents and caregivers themselves who are navigating this developmental milestone. In on-going research, we are interviewing adolescents and caregivers to understand their perspectives on transition planning, licensure, driving readiness, barriers, and resource needs. Finally, while healthcare providers are being called upon by patients and families to support this developmental transition, our results highlight the urgent need for resources to be widely disseminated and made easily accessible to reduce barriers experienced by providers in assessing readiness, providing counseling, and offering connection to reliable resources.

## Data Availability

The data that support the findings of this study are available from the corresponding author, Rachel Myers, upon reasonable request.
